# Structural basis for severe pain caused by mutations in the S4-S5 linkers of voltage-gated sodium channel Na_V_1.7

**DOI:** 10.1073/pnas.2219624120

**Published:** 2023-03-30

**Authors:** Goragot Wisedchaisri, Tamer M. Gamal El-Din, Ning Zheng, William A. Catterall

**Affiliations:** ^a^Department of Pharmacology, University of Washington, Seattle, WA 98195; ^b^HHMI, University of Washington, Seattle, WA 98195

**Keywords:** sodium channel, action potential, channelopathies, pain, sensory nerves

## Abstract

Gain-of-function mutations in voltage-gated sodium channel Na_V_1.7 cause severe inherited pain syndromes, including inherited erythromelalgia (IEM). The structural basis of these disease mutations, however, remains elusive. Here, we focused on three mutations that all substitute threonine residues in the alpha-helical S4-S5 intracellular linker that connects the voltage sensor to the pore: Na_V_1.7/I234T, Na_V_1.7/I848T, and Na_V_1.7/S241T in order of their positions in the amino acid sequence within the S4-S5 linkers. Introduction of these IEM mutations into the ancestral bacterial sodium channel Na_V_Ab recapitulated the pathogenic gain-of-function of these mutants by inducing a negative shift in the voltage dependence of activation and slowing the kinetics of inactivation. Remarkably, our structural analysis reveals a common mechanism of action among the three mutations, in which the mutant threonine residues create new hydrogen bonds between the S4-S5 linker and the pore-lining S5 or S6 segment in the pore module. Because the S4-S5 linkers couple voltage sensor movements to pore opening, these newly formed hydrogen bonds would stabilize the activated state substantially and thereby promote the 8 to 18 mV negative shift in the voltage dependence of activation that is characteristic of the Na_V_1.7 IEM mutants. Our results provide key structural insights into how IEM mutations in the S4-S5 linkers may cause hyperexcitability of Na_V_1.7 and lead to severe pain in this debilitating disease.

## Significance

Gain-of-function mutations in voltage-gated sodium channel Na_V_1.7 cause the severe inherited pain syndrome inherited erythromelalgia (IEM). We introduced three IEM mutations into the ancestral bacterial sodium channel Na_V_Ab, which substitutes threonine residues in the S4-S5 linker connecting the voltage sensor to the pore. These mutants recapitulated the pathogenic negative shift in voltage dependence of activation. Structural analysis revealed a common mechanism, in which new hydrogen bonds are formed between the S4-S5 linker and the pore. Because the S4-S5 linkers couple voltage sensor movements to pore opening, these newly formed hydrogen bonds would stabilize the activated state and promote hyperexcitability. These results provide key structural insights into how IEM mutations in the S4-S5 linkers may cause severe pain in IEM.

Voltage-gated sodium (Na_V_) channels initiate and propagate action potentials in nerves and muscles (1, 2), and mutations in Na_V_ channels lead to many diseases of hyperexcitability (3, 4). Na_V_1.7 is expressed mainly in peripheral somatic and visceral sensory neurons in dorsal root ganglia and in sympathetic ganglion neurons, where it serves as a threshold channel that sets the gain of nociceptors for generation of pain signals (4, 5). Its slow rate of closed-state inactivation allows smaller and slower depolarizations to activate the channel and thereby enhances responses to small nociceptive stimuli (4, 6).

Neuropathic pain syndromes affect millions of people and cost billions of dollars in health care expenditure (7, 8). They arise from damage in peripheral nerves from injury, diabetes, autoimmune disease, and cancer chemotherapy. However, rare inherited or sporadic pain syndromes are caused by mutations in Na_V_1.7. Inherited erythromelalgia (IEM), paroxysmal extreme pain disorder, and small fiber neuropathy are caused by heterozygous gain-of-function mutations in Na_V_1.7 (4). Patients with these syndromes have episodes of intense pain, redness, and swelling in different parts of the body. No targeted treatment for these pain syndromes is available, but some pharmacological interventions are effective in selected individuals (9). On the other hand, homozygous loss-of-function mutations caused by gene truncation or deletion of Na_V_1.7 produce Congenital Insensitivity to Pain (CIP), a rare disorder in which patients do not feel pain despite having injuries (10). As a result, Na_V_1.7 is an attractive drug target for novel pain treatments (8), but the development of effective analgesics targeting Na_V_1.7 has been fraught with difficulties (11).

Na_V_1.7 belongs to the family of nine human Na_V_ channel subtypes (Na_V_1.1-Na_V_1.9), which are expressed in different excitable tissues (12, 13). Na_V_ channels contain 24 transmembrane (TM) segments that form four homologous, but not identical, 6-TM domains (*D*I to *D*IV). The first four TM segments in each homologous 6-TM domain (S1 to S4) form the voltage-sensing module (VS), which is connected via the alpha-helical S4-S5 intracellular linker to the pore module (PM) formed by the S5 and S6 segments and the P loop between them. Structural studies of the homotetrameric bacterial sodium channel Na_V_Ab showed that these four homologous domains come together to form a functional channel with an ion-conducting pore at its center (13, 14). Depolarization activates Na_V_ channels according to the “sliding-helix model”, in which Arg or Lys gating charges in the S4 segment move outward toward the extracellular side of the membrane upon depolarization (15, 16). Outward S4 movement triggers a major conformational change in the S4-S5 linkers on the intracellular side of the membrane and induces the opening of the activation gate of the pore by bending and rotation of the intracellular ends of the S6 segments (17). Similar voltage-gating mechanisms have been proposed based on structural studies of the bacterial sodium channel NaChBac (16), the voltage-sensitive phosphatase CiVSP from the ancestral eukaryote *Ciona* (18), the two-pore-domain channel TPC from plants and mammals (19), the sea urchin hyperpolarization- and cyclic nucleotide-gated channel (20), the human proton channel (21), the human ether-a-go-go channel (22), and the mammalian sodium channel Na_V_1.7 (23–25).

IEM mutations shift the voltage dependence of activation to more negative membrane potentials, allowing smaller depolarizations to trigger an action potential (4). These disease mutations cluster in *D*I and *D*II. However, the molecular and structural basis for the pathogenic effects of mutations that result in pain phenotypes in patients remains unclear. Here, we have uncovered the structural basis for the gain-of-function phenotypes caused by three IEM mutations that substitute threonine (Thr) residues in the S4-S5 linkers of Na_V_1.7. Although the structure of Na_V_1.7 has been determined by cryo-EM (26, 27), progress toward resolving the structure with disease mutations at high resolution has been limited by challenges in obtaining sufficient quantity of Na_V_1.7 protein (28), intrinsic mobility of the VSs, and the limited resolution of the VS afforded by cryo-EM. To overcome these limitations, we introduced these IEM mutations into Na_V_Ab, a bacterial ancestor of mammalian Na_V_ channels, confirmed their functional effects in negatively shifting the voltage dependence of channel activation, and determined their structures at high resolution by X-ray crystallography (14). The backbone structures of Na_V_Ab and Na_V_1.7 are similar within <3 Å rmsd in their core transmembrane domains, which is identical within the limit of resolution of the cryo-EM structures (14, 26, 27). Key structural elements involved in the activation of human Na_V_1.7 are transferable to other sodium channels (14, 23, 24, 29). Each mutation is represented four times in the homotetrameric structure of Na_V_Ab, facilitating structure determination at high resolution by X-ray crystallography. This approach has revealed a common pathogenic mechanism in which the hydroxyl groups of substituted Thr residues form hydrogen bonds that stabilize the activated state of the voltage sensor and thereby negatively shift the voltage dependence of activation. Our results unveil an unexpected structural and physiological basis for Na_V_1.7 channelopathy caused by these three IEM mutations in the S4-S5 linker at near-atomic resolution.

## Results

### Function of Na_V_Ab with Na_V_1.7 IEM Mutations in the S4-S5 Linker.

**Fig. 1.**
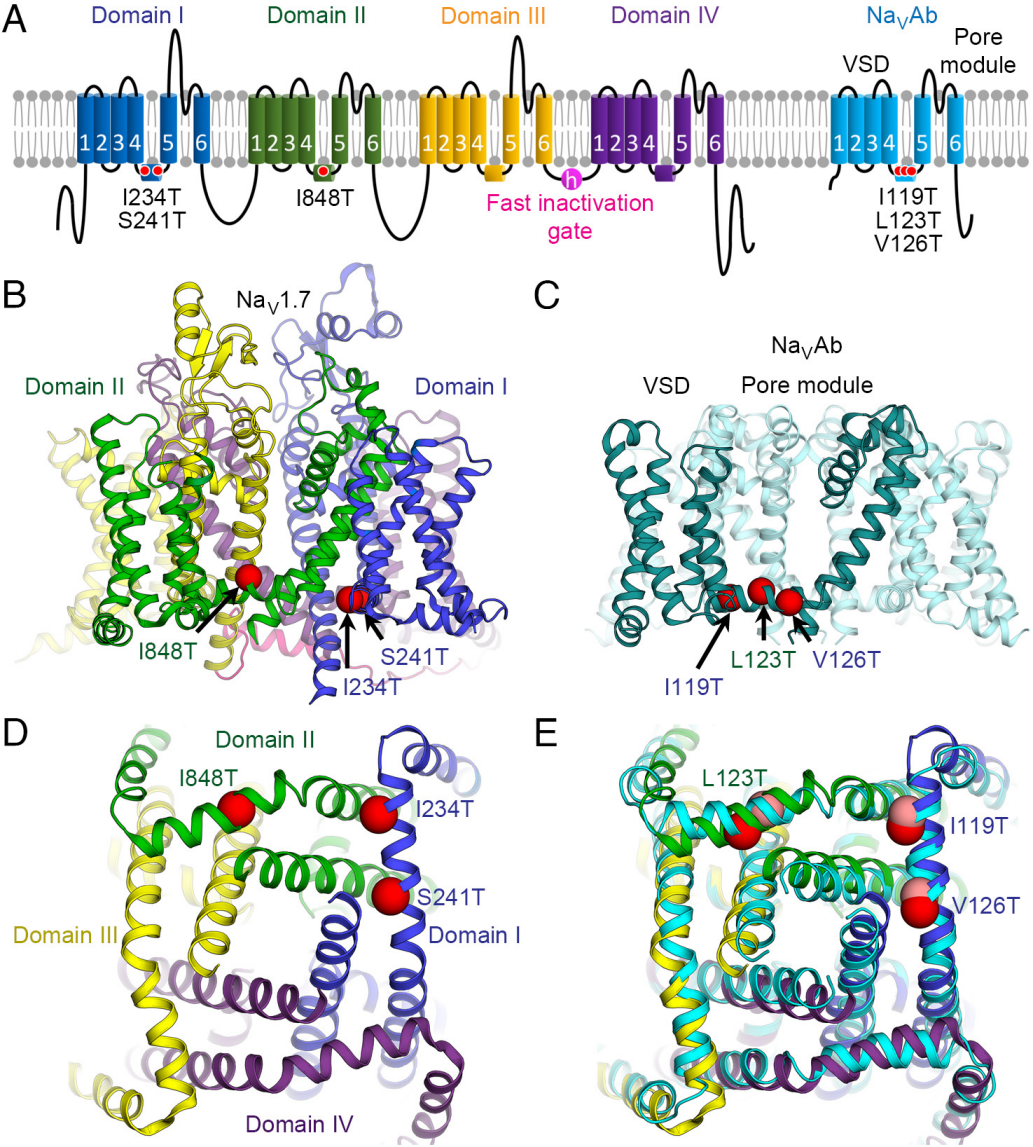
IEM mutations in voltage sensors of Na_V_1.7. (*A*) Topology diagram of Na_V_1.7 with seven IEM mutations (red circles) in the VSDs and the S4-S5 linkers of *D*I and *D*II selected for this study. Equivalent IEM mutations were introduced into the homotetrameric bacterial channel Na_V_Ab (cyan) for electrophysiological and structural characterization. (*B*) Structure of human Na_V_1.7 (PDB: 6J8I) with the locations of three IEM mutations mapped onto the model (red spheres). Each domain is colored as in *A*. (*C*) Structure of Na_V_Ab (PDB: 3RVY) with the locations of three IEM mutations mapped onto the model (red spheres). For clarity, the IEM mutations are shown only in one subunit (teal). Due to the tetrameric nature of Na_V_Ab, the mutations are also present in the remaining subunits (cyan). Residues are labeled with domain colors of their equivalent mutations in Na_V_1.7 as in *B*. (*D*) View of IEM mutations (red spheres) in Na_V_1.7 from the cytosolic side. (*E*) View of IEM mutations (red spheres) in Na_V_Ab from the cytosolic side. The structure of Na_V_1.7 from panel *D* is superimposed (with the IEM mutations shown as pink spheres) with an rmsd of ~2.0 Å for Cα atoms in the S4-S5 linkers and PM.

We surveyed IEM mutations in human Na_V_1.7 in the literature and selected three mutations that all substitute Thr residues in the S4-S5 linkers. These three IEM mutations are located in the alpha-helical S4-S5 linkers that connect the VS and PM: I234T and S241T in Na_V_1.7 *D*I and I848T in *D*II, which are equivalent to Na_V_Ab I119T, V126T, and L123T, respectively (Fig. 1*A*). The positions of these mutations in the S4-S5 linkers are illustrated in the side views of Na_V_1.7 and Na_V_Ab in Fig. 1 *B* and *C*, respectively. As viewed from the cytosolic side, the S4-S5 linkers of Na_V_1.7 surround the activation gate formed by the intracellular ends of the S6 segments (Fig. 1*D*). They are located in analogous positions in each of the four S4-S5 linkers in Na_V_Ab (Fig. 1*E*). The genetic background, phenotypic properties and biophysical effects as determined in previous studies of these mutations in Na_V_1.7 are summarized in *SI Appendix*, Tables S1 and S2.

**Fig. 2.**
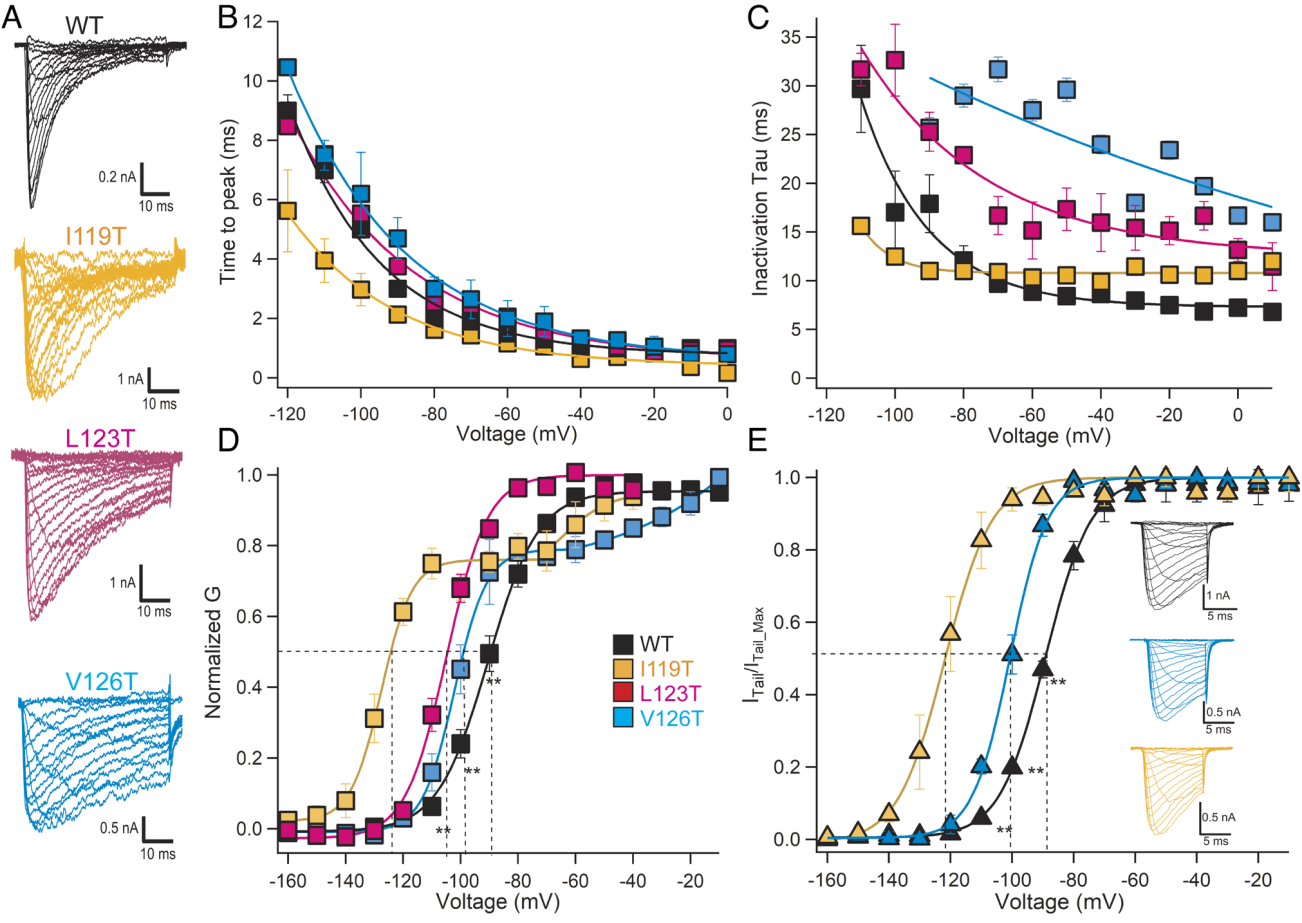
Functional properties of Na_V_Ab with IEM mutations. (*A*) Families of sodium currents. Na_V_Ab/Δ28 WT and mutants were expressed in Hi5 insect cells and studied by whole-cell voltage clamp recording as described in *Materials and Methods*. (*B*) Time to reach peak current is plotted as a function of stimulus voltage as calculated from the results of panel *A*. (*C*) Mean time constants for inactivation calculated from the results of experiments similar to those in panel *A* as described in *Materials and Methods*. (*D*) Voltage dependence of activation of WT and the indicated IEM mutants in the S4-S5 linker. Current/voltage (I/V) relationships of the peak sodium currents were recorded in response to steps to voltages ranging from −160 mV to +60 mV in 10-mV increments from a holding potential of −160 mV or −180 mV. Conductance/voltage (G/V) relationships were calculated as described in *Materials and Methods*. (*E*) Voltage-dependent activation curves determined from peak tail current amplitudes measured at −180 mV. Short depolarizing pulses, 10 ms, were applied from a holding potential of −180 mV in 10-mV steps. Tail currents were normalized to the highest amplitudes. (*Inset*) Representative traces of inward sodium currents and tail currents for WT, I119T, and V126T.

We introduced each of these S4-S5 linker IEM mutations into the C-terminal-truncated construct Na_V_AbΔ28, which was then expressed and analyzed by whole-cell voltage clamp (30; *Materials and Methods*). All of these constructs conduct voltage-gated inward sodium currents that activate rapidly and inactivate more slowly than wild-type (WT) Na_V_AbΔ28 (Fig. 2 *A*–*C*). Their rates of activation are equal to or more rapid than Na_V_Ab/WT in the critical voltage range from −50 mV to +10 mV, as illustrated in the plots of time-to-peak current vs. voltage in Fig. 2*B*. Similarly, these mutations increase the time constants for inactivation in the physiologically important voltage range of −60 to +10 mV (Fig. 2*C*), consistent with their hyperexcitability phenotype. In addition, the voltage dependence of activation (V_a_) for these mutants is negatively shifted to different extents as expected for IEM mutations (Fig. 2*D*). Surprisingly, the conductance/voltage (G/V) curves for mutants I119T and V126T are biphasic (Fig. 2*D*). Peak conductance in these G/V experiments is reached in 1 ms or less, raising the possibility that the voltage-dependent activation process of Na_V_Ab has not reached steady state in that short time. Therefore, we repeated steady-state activation experiments for these two mutants by depolarizing to a series of test voltages for 10 ms, hyperpolarizing to a fixed negative voltage (−180 mV), and measuring the peaks of the resulting inward tail currents as a measure of pore opening (Fig. 2*E*). The resulting tail current G/V curves are monophasic and show a clear negative shift of V_a_, consistent with the hypothesis that these mutants do indeed require 1 ms to 10 ms of depolarization to reach steady-state activation. We propose a structural basis for these unexpected findings in the Discussion. Overall, the rapid activation, slowed inactivation, and negatively shifted V_a_ of these mutants are consistent with their hyperexcitability phenotype in native Na_V_1.7 in vitro in transfected cells and in vivo in affected individuals.

### Structures of Na_V_Ab with IEM Mutations in S4-S5 Linkers.

We solubilized, purified, and crystallized the mutant Na_V_Ab proteins. All structures were determined for channels embedded in lipid bicelles by X-ray crystallography at a resolution ranging from 2.7 Å to 3.1 Å, which yielded near atomic clarity in these cases (*SI Appendix,* Table S3). All of these structures have their VSs in the activated state, as determined by the outward position of the gating charges in the S4 segment, while the PM is closed at the activation gate formed by the intracellular ends of the S6 segments. Because the IEM mutations primarily affect the activation process, we also generated structural models of the Na_V_Ab IEM mutants in the resting state, using the disulfide-crosslinked resting-state structure of Na_V_Ab as a template (17). This gave insight into the conformational transitions that are altered by the IEM mutations during activation of the VS. Notably, all three of these amino acid substitutions contain hydroxyl side chains of Thr residues that form new hydrogen bonds in our structures, which would stabilize the voltage sensor in its activated conformation. In the sections below, we consider these three IEM mutations in their order in the amino acid sequence of the S4-S5 linker of Na_V_Ab: I119T, L123T, and V126T (Fig. 1*C*).

### Na_V_Ab/I119T (Na_V_1.7/I234T).

Na_V_Ab/Ile119 is analogous to Ile234 in the S4-S5 linker in *D*I of Na_V_1.7, which is moderately conserved by replacement with an amino acid residue with a hydrophobic side chain of medium size in all four domains (*SI Appendix*, Fig. S1). Na_V_Ab/I119T shows a surprisingly large shift of −37.5 mV in V_a_ (Fig. 2 *D* and *E* and *SI Appendix,* Table S2). This shift of V_a_ is greater than the shift of −17.9 mV for Na_V_1.7/I234T, which was the largest negative shift among all IEM mutations in Na_V_1.7 (31).

**Fig. 3.**
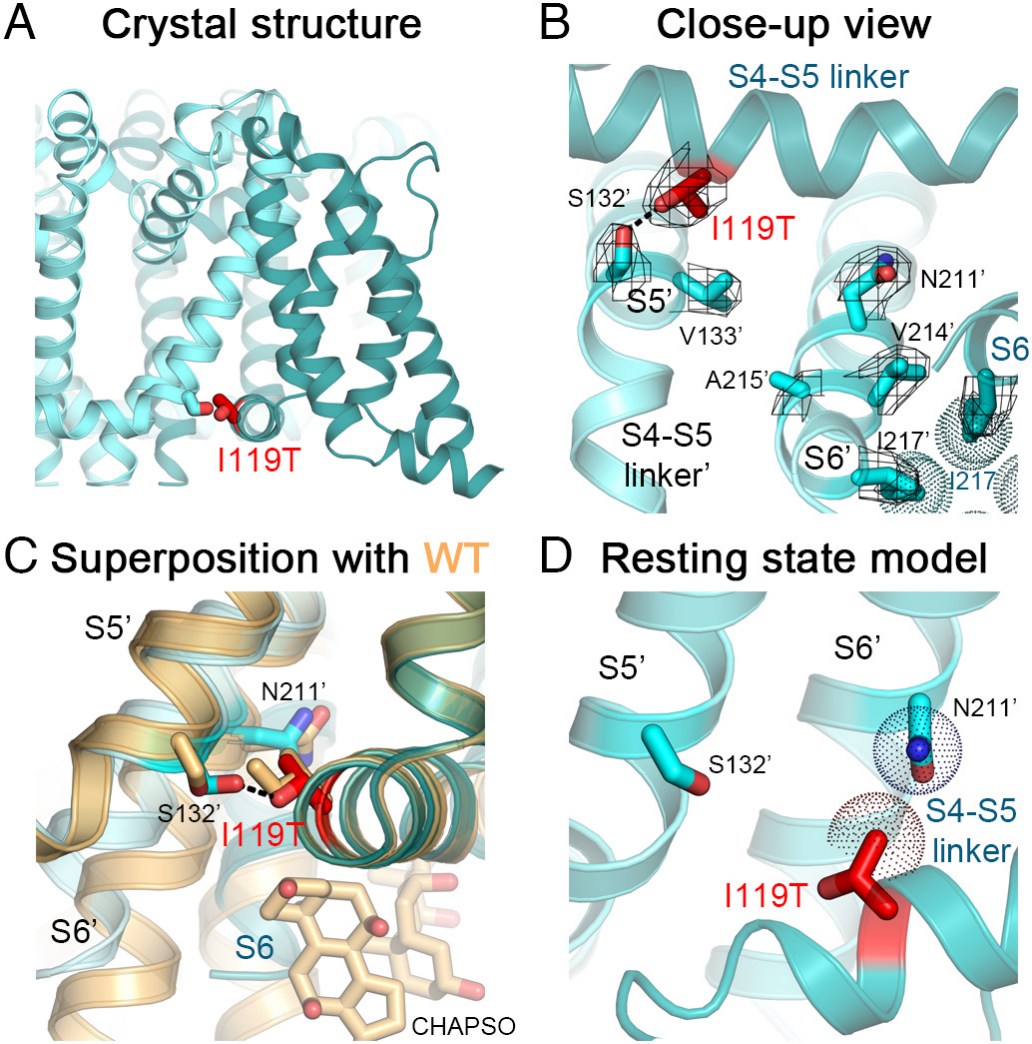
Structure of Na_V_Ab/I119T. (*A*) Crystal structure of Na_V_Ab/I119T with the mutation shown. (*B*) Close-up view of the Na_V_Ab/I119T structure with the VS in the activated state (teal and cyan for different subunits). Side chains of residues I119T (red), I217 (teal) and key side chains in S5 and S6 of a neighboring subunit (cyan) including Ser132′ and Asn211′ are shown as sticks with the σ_A_-weighted 2F_O_-F_C_ electron density map contoured at 1.0σ level overlaid (mesh). (*C*) Comparison of Na_V_Ab/I119T structure with the structure of Na_V_Ab WT (light orange). Side chain of I119T forms a hydrogen bond (black dashes) with side chain of Ser132′. Conformational changes in the C-terminal end of the S6 segments starting from Asn211′ in the mutant structure abolish the CHAPSO binding site present in the WT structure. (*D*) Homology model of Na_V_Ab/I119T structure in the resting state. The *van der Waals* hemispheres are shown as dots. Na_V_AbΔ28 (PDB: 6MWA) was used for the comparison.

The structure of Na_V_Ab/I119T at 2.75-Å resolution (Fig. 3*A*) clearly indicates that the side chain of I119T forms a hydrogen bond with Ser132 on S5 of a neighboring subunit (referred to as Ser132′) (Fig. 3 *A* and *B*). Although the overall structure is similar to WT (Cα rmsd = 0.7 Å), the conformations of the hinge region between the S4-S5 linker and the S5 segment where S132′ is located, as well as the activation gate receptor region of the pore-lining S6 segment, differ from the WT structure significantly (Fig. 3*C*). In the S4-S5 linker/S5 hinge region, the Cα atoms of Ile119 and Ser132′ are ~8 Å apart in the WT structure (Fig. 3*C*). This distance is shortened to ~7 Å in the I119T mutant structure due to the formation of a strong hydrogen bond (~2.5 Å) between the oxygen atoms of the I119T and Ser132′ side chains (Fig. 3*C*). Notably, the methyl group of the Thr side chain retains the hydrophobic interaction of Ile119 with Val133 in S5, further strengthening the interaction of these two side chains (Fig. 3*B*).

In human Na_V_1.7, Asn857 in *D*II S5 is the equivalent residue to Na_V_Ab Ser132′; therefore, the I234T mutation in the S4-S5 linker of *D*I of Na_V_1.7 is well positioned to form a hydrogen bond with the side chain of Asn857 in *D*II-S5 in the activated state, similar to Na_V_Ab/Ser132′. This newly formed hydrogen bond would stabilize the conformation of the activated state and thereby contribute to the negative shift in V_a_.

The S6 segment structure also exhibits a conformational change as it approaches the activation gate in Na_V_Ab/I119T, which starts with a kink around the conserved Asn211 residue and propagates by a rotation that causes a larger shift of ~4 Å at the last residue of S6 (Ala220) (Fig. 3*C*). This structural change may be induced by the formation of the hydrogen bond between I119T and Ser132′ and may contribute to its stabilization of the activated state and its unusually large negative shift of activation. The conformational change observed here disrupts two binding sites for CHAPSO detergent molecules from the bicelles that normally wedge between the S4-S5 linker and the pore-lining S6 segment in the WT structure, such that no electron density for the CHAPSO molecules was present in the mutant structure. Despite the conformational change in the activation gate, the pore structure of the mutant remains closed, with Ile217 forming a constriction site that seals the activation gate of the pore (Fig. 3*B*), similar to the WT structure.

The resting-state model of Na_V_Ab/I119T suggests that the I119T side chain forms weaker *van der Waals* interactions with the outer surface of the pore module from the neighboring subunit. Notably, the I119T side chain makes *van der Waals* contacts with the conserved Asn211′ residue from S6 with a distance of ~4 Å (Fig. 3*D*), similar to that for the native Ile residue found in the resting state of Na_V_Ab. Therefore, there is no evidence for a significant gain in stability by this mutation in the resting state. The gain of the hydrogen bond in the activated state from the I119T mutation, plus the conformational change induced in the S6 segment along with hydrogen bond formation, likely provides extra free energy that stabilizes the activated state and leads to channel hyperexcitability (Movie S1).

### Na_V_Ab/L123T (Na_V_1.7/I848T).

Na_V_Ab/Leu123 is analogous to Ile848 in *D*II of Na_V_1.7, which is conservatively replaced by Leu in the other three domains of Na_V_1.7 (*SI Appendix,* Fig. S1). This conservation of Leu and Ile residues suggests an important role for a hydrophobic side chain at this position. Electrophysiological recordings of Na_V_Ab/L123T show a shift of −15.5 mV in V_a_ (Fig. 2*D* and *SI Appendix*, Table S2), similar to negative shifts in V_a_ of −13.8 mV to −7.5 mV in previous studies of Na_V_1.7/I848T (32–34).

**Fig. 4.**
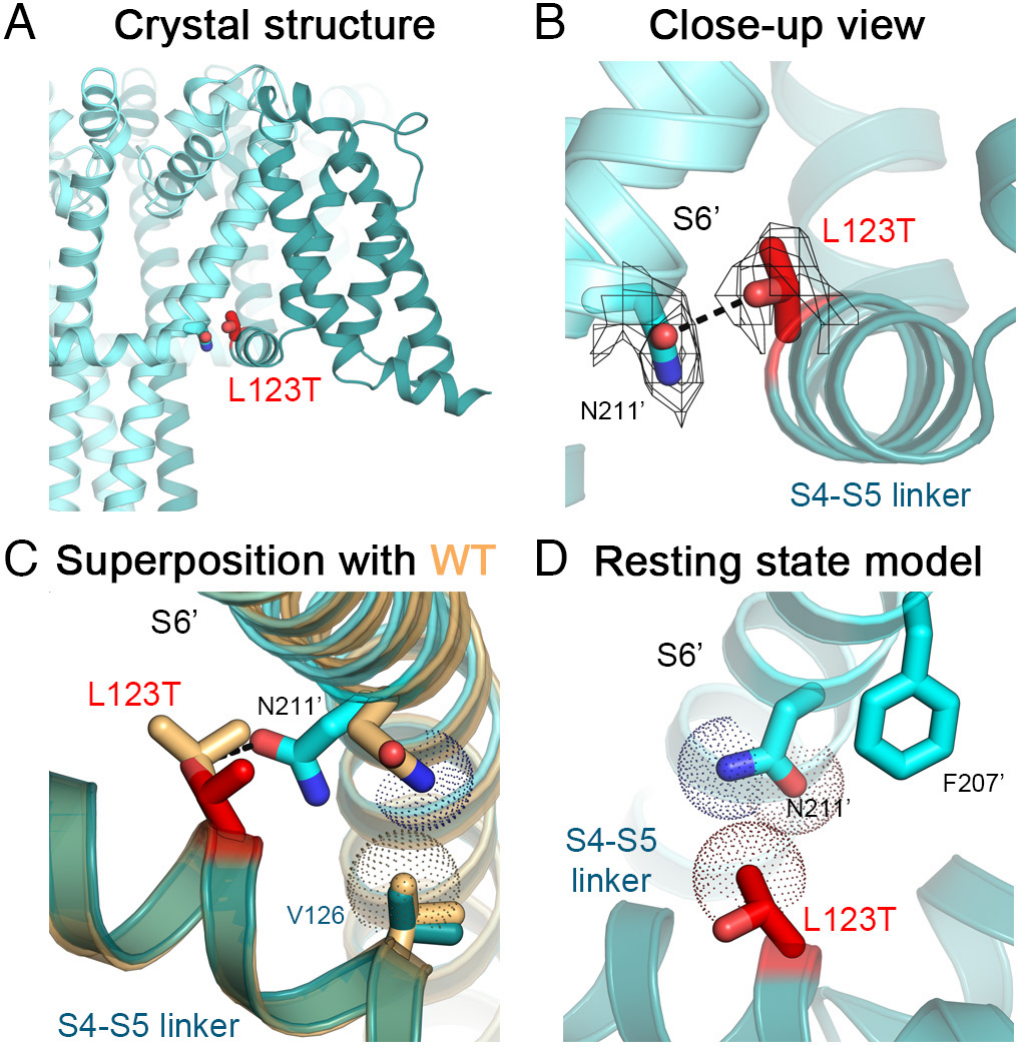
Structure of Na_V_Ab/L123T. (*A*) Crystal structure of Na_V_Ab/L123T with the mutation shown. (*B*) Close-up view of Na_V_Ab/L123T structure with the VS in the activated state (teal and cyan for different subunits). Side chains of residues L123T (red) and Asn211′ (cyan) are shown as sticks with the σ_A_-weighted 2F_O_-F_C_ electron density map contoured at 1.0σ level overlaid (mesh). (*C*) Comparison of Na_V_Ab/L123T structure with the structure of Na_V_Ab WT (light orange). Side chain of N211′ makes a *van der Waals* contact with the side chain of V126 in the WT structure but rotates to form a hydrogen bond (black dashes) with side chain of L123T in the mutant structure. The *van der Waals* hemispheres are shown as dots. (*D*) Homology model of Na_V_Ab/L123T structure in the resting state.

The structure of Na_V_Ab/L123T in the activated state at 2.7-Å resolution indicates that the substituted Thr side chain forms a hydrogen bond with the highly conserved Asn211 side chain in the S6 segment of a neighboring subunit (referred to as Asn211′) (Fig. 4 *A* and *B*). Asn211 is a conserved residue that is important for electromechanical coupling and critical for the gating process by bridging interactions between the S4-S5 linker and the S6 activation gate (17). In the WT structure, Asn211′ forms part of the wall of the ion-conducting pore with its side chain pointing toward the lumen, while also interacting with Val126 in the S4-S5 linker (Fig. 4*C*). In the L123T structure, the Asn211′ side chain adopts a different rotamer that points away from the lumen of the pore toward the S4-S5 linker and forms a hydrogen bond with a distance of ~3.1 Å to the hydroxyl oxygen of the L123T side chain (Fig. 4 *B* and *C*). The hydrogen bond formed between these two residues from the S4-S5 linker and the S6′ segment would provide additional stabilization of the activated state and therefore significantly shift voltage-dependent activation to more negative membrane potentials.

Modeling Na_V_Ab/L123T in the resting state suggests that the side chain of L123T makes *van der Waals* interactions with the conserved Asn211' side chain of an adjacent subunit (Fig. 4*D*). In contrast, the native Leu side chain in the Na_V_Ab resting state makes an additional hydrophobic interaction with the conserved Phe207’ residue located one helical turn from Asn211′. This interaction is absent in the L123T structure as the length of the side chain is reduced by the mutation, suggesting destabilization of the resting state by this mutation.

In human Na_V_1.7, Phe1435 and Asn1439 in *D*III S6 are found at the position equivalent to Na_V_Ab/Phe207 and Na_V_Ab/Asn211, respectively. Similarly, the I848T mutation likely abolishes the hydrophobic interaction with Phe1435 in the resting state, while creating a new hydrogen bond with Asn1439 in the activated state. Therefore, the loss of the hydrophobic interaction in the resting state and the gain of a hydrogen bond in the activated state both likely contribute to the large negative shift in V_a_ (Movie S2).

### Na_V_Ab/V126T (Na_V_1.7/S241T).

Na_V_Ab/Val126 is analogous to Na_V_1.7/Ser241. It is conserved in *D*I, *D*II, and *D*IV of Na_V_1.7 but is replaced by Ala in *D*III (*SI Appendix*, Fig. S1). Electrophysiological recordings of Na_V_Ab/V126T showed a shift of −12 mV in V_a_ (Fig. 2 *D* and *E* and *SI Appendix,* Table S2), similar to the shift of −8.4 mV for Na_V_1.7/S241T (35). These results indicate that Na_V_Ab/V126T is a valid structural model for this IEM mutation.

**Fig. 5.**
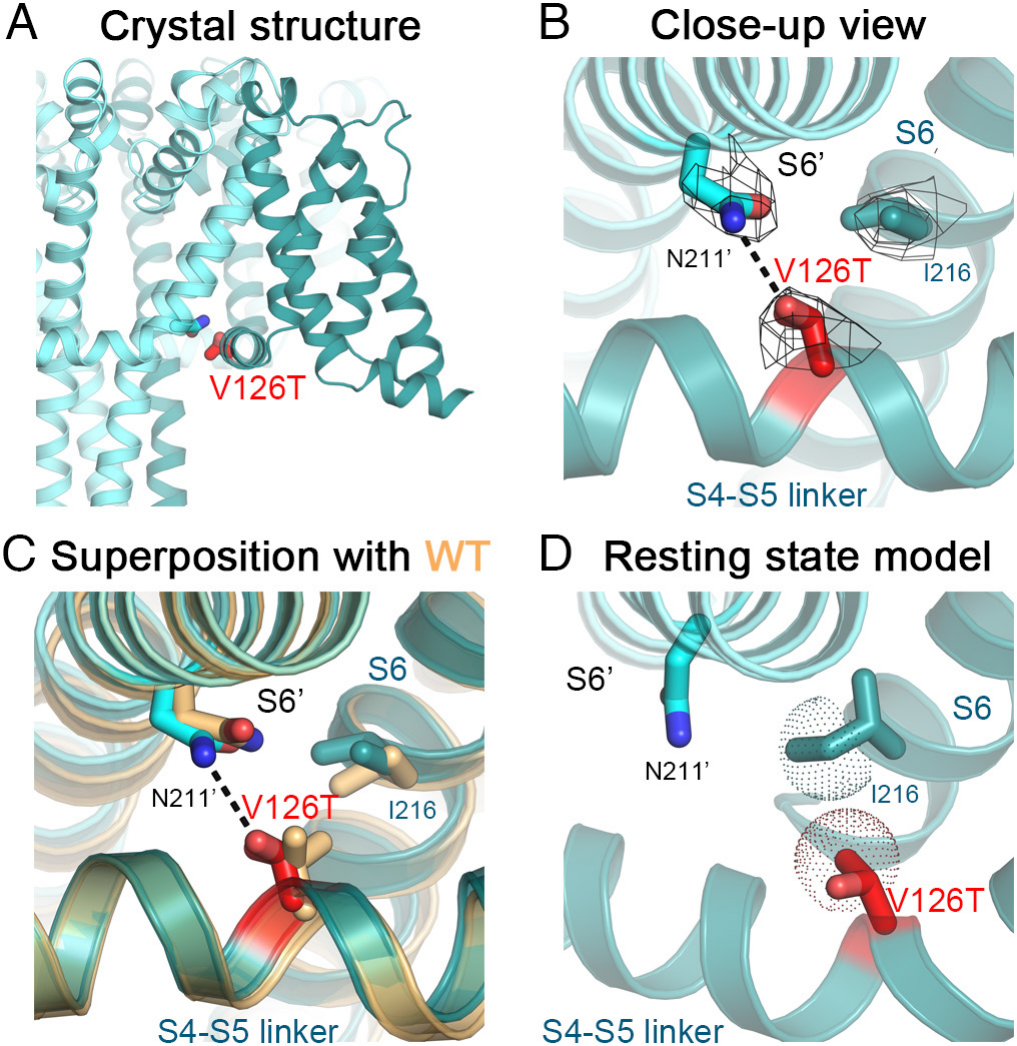
Structure of Na_V_Ab/V126T. (*A*) Crystal structure of Na_V_Ab/V126T with the mutation shown. (*B*) Close-up view of Na_V_Ab/V126T structure with the VS in the activated state (teal and cyan for different subunits). Side chains of residues V126T (red), Ile216 (teal), and Asn211’ (cyan) are shown as sticks with the σ_A_-weighted 2F_O_-F_C_ electron density map contoured at 1.0σ level overlaid (mesh). (*C*) Comparison of Na_V_Ab/V126T structure with the structure of Na_V_Ab WT (light orange). Side chain of V126T rotates to form a hydrogen bond (black dashes) with side chain of Asn211’. (*D*) Homology model of Na_V_Ab/L123T structure in the resting state. The *van der Waals* hemispheres are shown as dots.

The crystal structure of Na_V_Ab/V126T at 3.1-Å resolution (Fig. 5*A*) suggests that the V126T side chain forms a long-range hydrogen bond with the side chain of the Asn211′ residue in the S6 segment of a neighboring subunit (Fig. 5*B*). The V126T side chain rotates almost 180° from the Val side chain in the WT structure, twisting around the axis of the C_α_–C_β_ bond to reorient the oxygen atom toward the Asn211′ side chain with a distance of ~3.4 Å while maintaining *van der Waals* interaction with the side chain of Ile216 on S6 (Fig. 5*C*). In the WT structure, Val126 interacts with Asn211′ through *van der Waals* interactions with a distance of ~4 Å. The V126T mutation changes the chemistry of the interaction to a hydrogen bond while preserving the volume of the side chain and the hydrophobic interaction with Ile216. The hydrogen bond between Val126T and Asn211, which is absent in the resting-state homology model (Fig. 5*D* and (17)), likely enhances activation by providing additional stability to the activated state and thereby leads to channel hyperexcitability (Movie S3).

### Modeling the Mechanism of Action of IEM Mutations in Na_V_1.7 Channels.

**Fig. 6.**
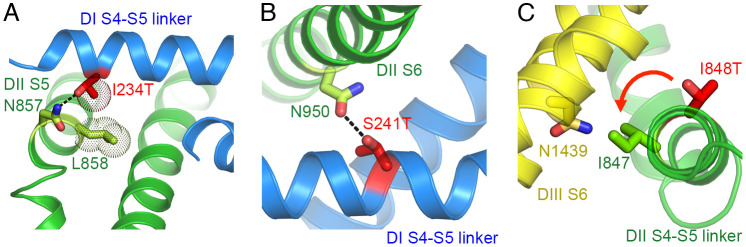
Potential hydrogen bond formation between mutant IEM residues in the S4-S5 linker and the PM of Na_V_1.7. (*A*) Na_V_1.7/I234T. (*B*) Na_V_1.7/S241T. (*C*) Na_V_1.7/I848T.

Na_V_1.7 has four distinct domains rather than four identical subunits as in Na_V_Ab. Similar mammalian Na_V_ channels have complex voltage-dependent gating mechanisms, typically involving four resting states, four resting/inactivated states, one open state, one fast inactivated state, and one or more slow inactivated states (36). The available cryo-EM structures of Na_V_1.7 are most likely to be in slow inactivated states, considering that the protein has been in a depolarized environment for an extended time (25, 26). Nevertheless, it is of interest to explore the potential functional relevance of the mechanism of action of the IEM mutations revealed here by modeling their impact on the available Na_V_1.7 structures (26) (Fig. 6). Of the three substituted Thr residues, we have studied, Na_V_1.7/I234T in the *D*I S4-S5 linker fits our proposed mechanism most closely. As in Na_V_Ab, the mutant Thr residue is predicted to form a high-energy hydrogen bond with nearby Asn857 in the *D*II S5 segment of Na_V_1.7, while maintaining *van der Waals* interaction with Leu858 (Fig. 6*A*). Similarly, the nearby residue Na_V_1.7/S241T is predicted to form a lower energy hydrogen bond with Asn950 in *D*II S6 of Na_V_1.7 (Fig. 6*B*). In contrast, Na_V_1.7/I848T in the S4-S5 linker in *D*II is rotated away from the potential hydrogen-bonding residue, Asn1439 in *D*III S6 in the available Na_V_1.7 structure (Fig. 6*C*). However, it is only one helical turn away from making this contact if the S4-S5 linker were to rotate counterclockwise by 60° to 90° from its position in the Na_V_1.7 inactivated state structure. Therefore, it is entirely possible that these two residues in Na_V_1.7 move near each other as the S4-S5 linker rotates during the activation process, similar to the small rotation of the S4-S5 linker in our gating model of Na_V_Ab (Movie S2 and (17)). This subtle movement would allow hydrogen bond formation to stabilize the activated state of Na_V_1.7.

## Discussion

Our study provides the first structural characterization of IEM mutations of human Na_V_1.7 channels and gives insight into the potential molecular basis for hyperexcitability in three sets of affected IEM families (*SI Appendix*, Table S1). We focused on the S4-S5 linkers of Na_V_1.7 where IEM mutations are clustered and coupling of voltage-dependent activation to pore opening takes place (15). By expressing these IEM mutations in the context of the ancestral sodium channel Na_V_Ab, we were able to isolate sufficient quantity of protein in the homogeneous form to allow crystallization and high-resolution structure determination of each mutation in its native state with an activated VS. In addition, because Na_V_Ab is the only sodium channel whose resting-state structure is known (17), we were able to use molecular modeling methods to define the effects of IEM mutations on the structures of both resting and activated states. By comparing the structural effects of these mutations in these two distinct states of the VS, a unified mechanism emerged for these three IEM mutants, which suggests a plausible structural basis for their hyperexcitability.

### IEM Mutations in the S4-S5 Linker Form Hydrogen Bonds with the Pore Module of Na_V_Ab.

The three IEM mutations we studied cause little or no change in the conformation of the S4-S5 linker. However, our structures indicate that these IEM mutations provide extra stability to the activated state by forming a new hydrogen bond with a residue from the PM of the adjacent subunit when the channel is in the activated state (Ser132 on S5 for I119T; Asn211 on S6 for L123T and V126T). All three of these mutated residues have been proposed to be important for electromechanical coupling and pore gating of the channel, based on the structure of Na_V_Ab in the resting and activated states (17). These hydrogen bonds are well-positioned to stabilize the conformation of the VS in the activated state. In contrast, these mutations do not form a hydrogen bond with any residue in the resting state, in which the S4-S5 linker protrudes as an elbow into the cytosol (17). The fact that mutations of these residues cause hyperexcitability that leads to severe pain in IEM further highlights the critical role of these residues in channel gating.

### IEM Mutations Differentially Stabilize the Activated States of Na_V_Ab and Na_V_1.7.

When we introduced these IEM mutations into Na_V_Ab, they caused negative shifts in V_a_ by −12 mV to −37.5 mV, with the mutation equivalent to I234T causing the largest change. For all three of these IEM mutations, the negative shift of V_a_ in Na_V_Ab is significantly greater than that for the same mutation in Na_V_1.7 (*SI Appendix*, Table S2). In the most prominent example, V_a_ for Na_V_Ab/I119T is −37.5 mV compared to −17.9 mV for V_a_ in Na_V_1.7/I234T (*SI Appendix*, Table S2). In comparison, the shift of V_a_ in Na_V_Ab/L123T is −15.5 mV compared to −10.4 mV (mean of n = 3 published studies; *SI Appendix*, Table S2) for V_a_ in Na_V_1.7/I848T, whereas the shift of V_a_ in Na_V_Ab/V126T is −12 mV compared to −8.4 mV for V_a_ in Na_V_1.7/S241T (*SI Appendix*, Table S2). It is likely that two distinct factors contribute to these differences in V_a_. First, the energy of hydrogen bonds diminishes with their length, and the bond lengths for Na_V_Ab/I119T, Na_V_Ab/L123T, and Na_V_Ab/V126T are approximately 2.5 Å, 3.1 Å, and 3.4 Å, respectively, providing a structural basis for their different shifts of V_a_ based on their hydrogen bond length. Second, we consider it likely that the shifts of V_a_ are larger in Na_V_Ab because of conformational coupling among the four copies of the mutation in the tetramer, and this coupling energy may be greater for Na_V_Ab/I119T than for the other two mutants.

### IEM Mutations Are Well-Positioned to Form Hydrogen Bonds in Na_V_1.7.

The I234T, S241T, and I848T mutations are located in the S4-S5 linkers in *D*I and *D*II of Na_V_1.7. Models of these mutations in the structure of Na_V_1.7 directly support the formation of hydrogen bonds in the activated state for two of these three mutations, and the third mutation is in position to form a potential new hydrogen bond with modest structural change during the activation process (Fig. 6). These newly formed hydrogen bonds could contribute up to 6 kcal/mol to stabilize the activated state in Na_V_1.7, which would make a substantial contribution to the free energy required for the 8 mV to 18 mV negative shifts in V_a_ of these mutants in Na_V_1.7 (*SI Appendix*, Table S2). Stabilization of the activated state would lead to voltage-sensor trapping, similar to the effects of neurotoxins that negatively shift V_a_ or block fast inactivation by binding with high affinity to the activated state of Na_V_ channels (37, 38). Voltage sensor trapping in the activated state leads to prolonged trains of action potentials, similar to those induced by IEM mutations (4, 31). Interestingly, recent studies of phosphorylation of Na_V_1.7/I848T also showed a negative shift of −9.8 mV in V_a_ mediated by Protein Kinase C (34), which would further enhance the negative shift of V_a_ caused by this mutation that we have observed in Na_V_Ab (ΔV_1/2_ of −15.5 mV). Thus, our high-resolution information on these focused structural changes induced by IEM mutations gives unprecedented insights into IEM pathogenesis at near-atomic resolution and provides a potential molecular template for mutation-specific therapy of this debilitating disease.

### Biphasic Kinetics of IEM Mutants Suggests Hydrogen Bond Formation During Activation.

Mutations Na_V_Ab/I119T and Na_V_Ab/V126T give two-component Boltzmann fits of their activation curves, as if the voltage sensor activates in two steps (Fig. 2*D*). These results raise the possibility that the formation of hydrogen bonds with Ser132' and Asn211' is incomplete, resulting in a major fraction of the channels that open with negatively shifted voltage dependence stabilized by hydrogen bond formation and a minor fraction of channels that do not form hydrogen bonds successfully during the brief stimulus pulses. Although our stimulus pulses were 50 ms in duration, actual measurements of peak sodium currents were made at stimulus times less than 1 ms at the most positive stimulus potentials (Fig. 2 *A*–*D*). To test whether these short stimulus durations are responsible for the biphasic activation curves, we measured G/V curves from tail currents. In this protocol, cells were depolarized to different membrane potentials during a prepulse of 10 ms, and inward sodium currents through the channels that have opened were measured during a hyperpolarizing pulse to −180 mV (Fig. 2*E*). During this protocol, the VS have up to 10 ms to activate at all stimulus potentials. Remarkably, the biphasic activation curves were resolved to approximately single Boltzmann fits with this protocol (Fig. 2*E*), confirming that biphasic activation curves were caused by the short times available to reach peak current in the standard G/V curve protocol. This striking feature of our data provides further support for the importance of hydrogen bond formation to stabilize the activated state of the VS and cause a negative shift in V_a_ and further suggests that these amino acid side chains can align and hydrogen bonds can form completely in less than 10 ms.

### Slow Inactivation of IEM Mutants in Na_V_Ab.

Mutations that cause IEM have variable effects on slow inactivation of Na_V_1.7 channels, suggesting that these effects are not central to the IEM phenotype but may modify it in individuals with specific mutations (4, 7). Slow inactivation of mammalian sodium channels involves conformational movements of the outer pore region (39–41). Bacterial Na_V_ channels have a slow inactivation process that causes pore closure, similar to the slow inactivation of mammalian Na_V_ channels, and analogous to conformational changes in transmembrane closure of the pore in the slow inactivation of the bacterial potassium channel KcsA (42–45). Na_V_Ab has a complex three-phase mode of slow inactivation involving movements of the selectivity filter and outer pore as well as asymmetric closure of the intracellular activation gate formed by the S6 segments (30, 44). Consistent with previous studies, we found that the IEM mutations we studied here primarily slowed the rate of inactivation in the physiologically important membrane potential range from −60 mV to 0 mV (Fig. 2*C*). These effects on slow inactivation of IEM mutations expressed in Na_V_Ab are consistent with their hyperexcitable phenotype in vivo. However, individual IEM mutations have a range of effects from little or no change to substantial delay of slow inactivation during trains of action potentials (4, 7); therefore, they probably do not contribute in a major way to the disease phenotype in most individuals with mutations in human Na_V_1.7 (46). Negatively shifted V_a_, stabilized by the formation of new hydrogen bonds, is most likely to be the underlying mechanism for hyperexcitability for the IEM mutations studied here.

## Materials and Methods

### Materials Availability.

Further information and requests for resources and reagents should be directed to the corresponding author William A. Catterall (wcatt@uw.edu). All reagents generated in this study are available from the corresponding author with a completed Materials Transfer Agreement.

### Microbe Strains.

*Escherichia coli* GC10 was cultured at 37 °C in an LB medium supplemented with 100 mg/mL of ampicillin for plasmid DNA extraction. *E. coli* DH10Bac was cultured at 37 °C in LB medium supplemented with 50 mg/mL kanamycin sulfate, 7 mg/mL gentamicin, and 10 mg/mL tetracycline for bacmid production.

### Cell Lines.

Sf9 (*Spodoptera frugiperda*) insect cells were maintained in Grace’s Insect Medium and supplemented with 8 to 10% fetal bovine serum (FBS) and penicillin/streptomycin at 27 °C and passaged at 80 to 95% confluence for baculovirus production. Hi5 (*Trichoplusia ni*) insect cells were maintained and infected in Grace’s Insect Medium supplemented with 8 to 10% FBS and glutamine/penicillin/streptomycin at 27 °C for electrophysiology and protein expression.

### Mutagenesis and Baculovirus Production of Na_V_Ab with IEM Mutations.

The pFastBac-Na_V_AbΔ28 plasmid carrying a C-terminal 28-residue truncation (residues 1 to 239) of Na_V_Ab gene (30) was used as a template for site-directed mutagenesis. To generate a desired plasmid of a Na_V_Ab mutant, overlapping oligonucleotide primers with a codon changed to a specific mutation were synthesized (Integrated DNA Technologies) and used for site-directed mutagenesis PCR with PfuUltra II Fusion HotStart DNA Polymerase (Agilent). The PCR products were treated with DpnI (New England Biolabs) and transformed into *E. coli* GC10 competent cells (Genesee Scientific). Plasmid DNAs were isolated from transformed colonies using the QIAprep Spin Miniprep Kit (Qiagen) according to the manufacturer’s protocol and sequenced to confirm the mutation. Plasmids containing Na_V_AbΔ28 with the IEM mutation were used to transform *E. coli* DH10Bac competent cells for bacmid production and baculoviruses were prepared with Sf9 insect cells using the Bac-to-Bac protocol according to the manufacturer (Life Technologies).

### Electrophysiology of Na_V_Ab with IEM Mutations.

Hi5 insect cells (*Trichoplusia ni*) were infected with baculovirus containing Na_V_AbΔ28 with an IEM mutation (30). After 24 to 48 h, whole-cell sodium currents were recorded using an Axopatch 200 amplifier (Molecular Devices) with glass micropipettes (1.5 to 2.5 MΩ) (30). The intracellular pipette solution contained (in mM): 35 NaCl, 105 CsF, 10 EGTA, 10 HEPES, pH 7.4 (adjusted with CsOH). The extracellular solution contained (in mM): 140 NaCl, 2 CaCl_2_, 2 MgCl_2_, 10 HEPES, pH 7.4 (adjusted with NaOH). Linear capacitance was subtracted and 80 to 90% of series resistance was compensated using internal amplifier circuitry. I/V relationships of the peak currents were recorded in response to steps to voltages ranging from −160 mV to +160 mV in 10-mV increments from a holding potential of −160 mV or −180 mV. In the case of voltage-dependent activation curves determined from peak tail current amplitudes measured at −180 mV, 10-ms depolarizing pulses were applied from a holding potential of −180 mV in 10-mV steps. Tail currents were normalized to the highest amplitudes. Pulses were generated and currents were recorded using Pulse software controlling an InstruTECH ITC-18 interface (HEKA). Data were analyzed using Igor Pro 8 (WaveMetrics).

### Analysis of Electrophysiological Data.

Voltage-clamp data were analyzed using IGOR Pro 8 (WaveMetrics). Peak current at each voltage of the current family was plotted as a function of the stimulus voltage to visualize the current *vs*. voltage (I/V) relationship. For WT and mutant L123T, normalized conductance/voltage (G/V) curves were calculated from the I/V curves and fit with a simple one-component Boltzmann equation: 1/(1+exp(V_a_−V_m_)/k) in which V_m_ is the stimulus potential, V_a_ is the half-activation voltage, and k is a slope factor. Similarly, for WT, I119T, and V126T, G/V curves were determined from peak tail current amplitudes by fit to this simple one-component Boltzmann relationship.

In contrast, biphasic G/V curves for I119T and V126T were calculated from I/V curves and fit with a two component Boltzmann equation:

$$y=yo+A\;\left[\frac{p}{1+{\text{e}}^{\frac{\text{Va}1-\text{Vm}}{k1}}}+\frac{1-p}{1+{\text{e}}^{\frac{\text{Va}2-\text{Vm}}{k2}}}\right]$$

where *y*_0_ and *A* are the minimum and the maximum values taken by *y*. *p* is the fraction of the curve comprising first sigmoidal curve, (1 − *p*) is the fraction of the curve comprising the second sigmoidal curve, and Va1, Va2, *k_1_*, and *k_2_* are the voltage midpoints and slope factors of the two phases. The results for the I119T mutation fit well to this two-component Boltzmann equation with Va1 = −127.6 mV, *p* = 0.76, *k1* = 5.34 mV, Va2 = −64 mV, 1-*p* = 0.24, and *k2 *= 8.3 mV. However, the double-sigmoidal curve did not fit well for the second phase of activation of the V126T mutation, so only the first component was fitted with Va = −102 mV, *k* = 5.6 mV.

The half-time (τ) for inactivation was measured from the peak of each current trace. A single exponential function was used to fit the inactivation kinetics of all three mutants.
The data were presented as mean and SEM. Statistical significance was evaluated with Student’s *t* test.

### Protein Expression and Purification of Na_V_Ab with IEM Mutations.

Third passage (P3) baculoviruses for Na_V_AbΔ28 with an IEM mutation were used to infect Hi5 cells in single-layer culture dishes (~12 dishes for each preparation), and the cells were incubated at 27 °C for ~72 h. The cells were harvested by centrifugation, and the pellets were resuspended in Buffer A (50 mM Tris HCl pH 7.5 and 200 mM NaCl) supplemented with 1 mM PMSF, 2× SigmaFast protease inhibitor cocktails (MilliporeSigma), benzamidine HCl, and DNase I. Cells were lysed by sonication and membranes were solubilized with 1% high-purity digitonin (MilliporeSigma) for 1 h at 4 °C with gentle mixing. The mixture was centrifuged at 15,000×g for 30 min at 4 °C, and the supernatant was incubated with Anti-FLAG-M2 affinity gel (MilliporeSigma) for 1 h at 4 °C with gentle mixing. The resins were washed with Buffer B (Buffer A supplemented with 0.12% digitonin) and bound protein was eluted with Buffer C (Buffer B supplemented with 200 μM FLAG peptide). Eluted protein was concentrated to 1 mL using Vivaspin20 100 kDa MWCO (Cytiva) and further purified with Superdex S200 size-exclusion chromatography (Cytiva) using 10 mM Tris HCl pH 7.5, 100 mM NaCl, and 0.12% digitonin as a column running buffer. Elution fractions were evaluated using SDS-PAGE, and peak fractions were combined and concentrated to final concentration of ~20 mg/ml. Protein concentrations were estimated using 1 A_280_ absorbance unit = 1 mg/mL on a NanoDrop spectrophotometer.

### Crystallization of Na_V_Ab with IEM Mutations.

Na_V_Ab-bicelle complexes were prepared by mixing Na_V_AbΔ28 with an IEM mutation with 10% bicelle (7.5% w/v DMPC and 2.5% w/v CHAPSO) at 1:4 or 1:5 volume ratios to obtain final 3.0 to 3.5 mg/ml protein concentration. The complexes were screened for crystallization conditions under 1.70 to 1.95 M ammonium sulfate and 0.1 M sodium citrate tribasic, pH 4.6 to 6.0 (Hampton Research). Crystals were cryo-protected by stepwise transfers to a series of cryo-protectant solutions containing 6 to 30% glucose (6% increments) with the same concentration of ammonium sulfate and sodium citrate in which the crystals were grown.

### X-ray Data Collection and Structure Determination of Na_V_Ab with IEM Mutations.

Crystals were tested for diffraction, and data were collected at Advanced Light Source beamline 8.2.1 and 8.2.2 Howard Hughes Medical Institute. Diffraction data were processed using the HKL2000 program (47) with anisotropic scaling and truncation due to anisotropic diffraction. Structures were solved by molecular replacement with PHASER (48) using the previously determined Na_V_Ab structure PDB: 3RVY (14) or PDB: 6MW (30) as a search model and refined with REFMAC (49) in the CCP4 program suite (50). Manual model building and local real space refinement were carried out in COOT (51), followed by structure refinement in REFMAC. Subsequently structures were refined with Phenix.refine module and the final structures were analyzed and validated using MolProbity in the Phenix program suite (52) (*SI Appendix*, Table S3).

## Modeling of Na_V_Ab Structure with IEM Mutations in the Resting State.

Homology models of Na_V_Ab tetramer structure with the IEM mutations in the resting state were generated with Modeller 10.2 (53) using the disulfide-locked Na_V_Ab/KAV/G94C/Q150C cryo-EM structure (PDB: 6P6W) (17) as a template. Structure figures and morph movies were made with Pymol (Schrodinger).

### Modeling of Na_V_1.7 Structure with IEM Mutations.

Cryo-EM structure of human Na_V_1.7 (PDB: 7W9K) was used for modeling in Coot (51). Residues with IEM mutations were mutated to threonine and the most probable side chain rotamer without clashes was selected. Different rotamers of residues that potentially form hydrogen bond pairs with the IEM mutations were tested and a rotamer within a hydrogen bond distance without clashes was selected.

## Supplementary Material

Appendix 01 (PDF)

Movie S1. Transition of Na_V_Ab/I119T from the resting state to the activated state from the intracellular view. I119T and Val126 are shown as red and orange sticks, respectively. Ser132’ and Asn211’ from a neighboring subunit are shown as yellow and cyan sticks, respectively. S4 is colored in magenta, the S4-S5 linker in orange, and the pore module S5 to S6 in cyan. Dashes indicate distance in Å.

Movie S2. Transition of Na_V_Ab/L123T from the resting state to the activated state from the intracellular view. L123T, and Asn211’ from a neighboring subunit are shown as red and cyan sticks, respectively. Dashes indicate distance in Å.

Movie S3. Transition of Na_V_Ab/V126T from the resting state to the activated state from the intracellular view. V126T, Ile216, and Asn211’ from a neighboring subunit are shown as red, teal, and cyan sticks, respectively. Dashes indicate distance in Å.

## Data Availability

The coordinates and the structure factors for the reported crystal structures have been deposited in the Protein Data Bank (PDB) under accession codes 8DIZ (54), 8DJ0 (55), and 8DJ1 (56) for Na_V_AbΔ28 I119T, L123T, and V126T, respectively. All study data are included in the article and/or *SI Appendix*.

## References

[1] A. L. Hodgkin, A. F. Huxley, A quantitative description of membrane current and its application to conduction and excitation in nerve. J. Physiol. **117**, 500–544 (1952). 12991237 10.1113/jphysiol.1952.sp004764 PMC1392413

[2] R. H. Adrian, W. K. Chandler, A. L. Hodgkin, Voltage clamp experiments in striated muscle fibres. J. Physiol. **208**, 607–644 (1970). 5499787 10.1113/jphysiol.1970.sp009139 PMC1348789

[3] M. Mantegazza, S. Cestèle, W. A. Catterall, Sodium channelopathies of skeletal muscle and brain. Physiol. Rev. **101**, 1633–1689 (2021). 33769100 10.1152/physrev.00025.2020 PMC8989381

[4] S. D. Dib-Hajj, Y. Yang, J. A. Black, S. G. Waxman, The Nav1.7 sodium channel from molecule to man. Nat. Rev. Neurosci. **14**, 49–62 (2013). 23232607 10.1038/nrn3404

[5] N. Klugbauer, L. Lacinova, V. Flockerzi, F. Hofmann, Structure and functional expression of a new member of the tetrodotoxin-sensitive voltage-activated sodium channel family from human neuroendocrine cells. EMBO J. **14**, 1084–1090 (1995). 7720699 10.1002/j.1460-2075.1995.tb07091.x PMC398185

[6] T. R. Cummins, J. R. Howe, S. G. Waxman, Slow closed-state inactivation: A novel mechanism underlying ramp currents in cells expressing the hNE/PN1 sodium channel. J. Neurosci. **18**, 9607–9619 (1998). 9822722 10.1523/JNEUROSCI.18-23-09607.1998 PMC6793269

[7] S. G. Waxman , Sodium channel genes in pain-related disorders: Phenotype-genotype associations and recommendations for clinical use. Lancet Neurol. **13**, 1152–1160 (2014). 25316021 10.1016/S1474-4422(14)70150-4

[8] S. D. Dib-Hajj, S. G. Waxman, Translational pain research: Lessons from genetics and genomics. Sci. Transl. Med. **6**, 249sr244 (2014). 10.1126/scitranslmed.3007017 25122641

[9] P. Geha , Pharmacotherapy for pain in a family with inherited erythromelalgia guided by genomic analysis and functional profiling. JAMA Neurol **73**, 659–667 (2016). 27088781 10.1001/jamaneurol.2016.0389

[10] J. J. Cox , An SCN9A channelopathy causes congenital inability to experience pain. Nature **444**, 894–898 (2006). 17167479 10.1038/nature05413 PMC7212082

[11] J. V. Mulcahy , Challenges and opportunities for therapeutics targeting the voltage-gated sodium channel Isoform Na_V_1.7. J. Med. Chem. **62**, 8695–8710 (2019). 31012583 10.1021/acs.jmedchem.8b01906 PMC6786914

[12] W. A. Catterall, From ionic currents to molecular mechanisms: The structure and function of voltage-gated sodium channels. Neuron **26**, 13–25 (2000). 10798388 10.1016/s0896-6273(00)81133-2

[13] C. A. Ahern, J. Payandeh, F. Bosmans, B. Chanda, The hitchhiker’s guide to the voltage-gated sodium channel galaxy. J. Gen. Physiol. **147**, 1–24 (2016). 26712848 10.1085/jgp.201511492 PMC4692491

[14] J. Payandeh, T. Scheuer, N. Zheng, W. A. Catterall, The crystal structure of a voltage-gated sodium channel. Nature **475**, 353–358 (2011). 21743477 10.1038/nature10238 PMC3266868

[15] W. A. Catterall, G. Wisedchaisri, N. Zheng, The chemical basis for electrical signaling. Nat. Chem. Biol. **13**, 455–463 (2017). 28406893 10.1038/nchembio.2353 PMC5464002

[16] V. Yarov-Yarovoy , Structural basis for gating charge movement in the voltage sensor of a sodium channel. Proc. Natl. Acad. Sci. U.S.A. **109**, E93–E102 (2012). 22160714 10.1073/pnas.1118434109 PMC3258622

[17] G. Wisedchaisri , Resting-state structure and gating mechanism of a voltage-gated sodium channel. Cell **178**, 993–1003.e1012 (2019). 31353218 10.1016/j.cell.2019.06.031 PMC6688928

[18] Q. Li , Structural mechanism of voltage-dependent gating in an isolated voltage-sensing domain. Nat. Struct. Mol. Biol. **21**, 244–252 (2014). 24487958 10.1038/nsmb.2768 PMC4116111

[19] M. S. Dickinson, A. Myasnikov, J. Eriksen, N. Poweleit, R. M. Stroud, Resting state structure of the hyperdepolarization activated two-pore channel 3. Proc. Natl. Acad. Sci. U.S.A. **117**, 1988–1993 (2020). 31924746 10.1073/pnas.1915144117 PMC6995003

[20] G. Dai, T. K. Aman, F. DiMaio, W. N. Zagotta, The HCN channel voltage sensor undergoes a large downward motion during hyperpolarization. Nat. Struct. Mol. Biol. **26**, 686–694 (2019). 31285608 10.1038/s41594-019-0259-1 PMC6692172

[21] Q. Li , Resting state of the human proton channel dimer in a lipid bilayer. Proc. Natl. Acad. Sci. U.S.A. **112**, E5926–5935 (2015). 26443860 10.1073/pnas.1515043112 PMC4640771

[22] V. S. Mandala, R. MacKinnon, Voltage-sensor movements in the Eag Kv channel under an applied electric field. Proc. Natl. Acad. Sci. U.S.A. **119**, e2214151119 (2022). 36331999 10.1073/pnas.2214151119 PMC9674223

[23] G. Wisedchaisri , Structural basis for high-affinity trapping of the Na_V_1.7 channel in its resting state by a tarantula toxin. Mol. Cell **81**, 38–48.e34 (2021). 33232657 10.1016/j.molcel.2020.10.039 PMC8043720

[24] H. Xu , Structural basis of Na_V_1.7 inhibition by a gating-modifier spider toxin. Cell **176**, 702–715.e14 (2019). 30661758 10.1016/j.cell.2018.12.018

[25] G. Huang , Unwinding and spiral sliding of S4 and domain rotation of VSD during the electromechanical coupling in Nav1.7. Proc. Natl. Acad. Sci. U.S.A. **119**, e2209164119 (2022). 35878056 10.1073/pnas.2209164119 PMC9388133

[26] H. Shen, D. Liu, K. Wu, J. Lei, N. Yan, Structures of human Na_V_1.7 channel in complex with auxiliary subunits and animal toxins. Science **363**, 1303–1308 (2019). 30765606 10.1126/science.aaw2493

[27] G. Huang , High-resolution structures of human Na_V_1.7 reveal gating modulation through α-π helical transition of S6_IV_. Cell Rep. **39**, 110735 (2022). 35476982 10.1016/j.celrep.2022.110735

[28] H. Shen, N. Yan, X. Pan, Structural determination of human Na_V_1.4 and Na_V_1.7 using single particle cryo-electron microscopy. Methods Enzymol. **653**, 103–120 (2021). 34099168 10.1016/bs.mie.2021.03.010

[29] S. Ahuja , Structural basis of Na_V_1.7 inhibition by an isoform-selective small-molecule antagonist. Science **350**, aac5464 (2015). 26680203 10.1126/science.aac5464

[30] T. M. Gamal El-Din, M. J. Lenaeus, K. Ramanadane, N. Zheng, W. A. Catterall, Molecular dissection of multiphase inactivation of the bacterial sodium channel Na_V_Ab. J. Gen. Physiol. **151**, 174–185 (2019). 30510035 10.1085/jgp.201711884 PMC6363407

[31] H. S. Ahn , A new Na_V_1.7 sodium channel mutation I234T in a child with severe pain. Eur. J. Pain **14**, 944–950 (2010). 20385509 10.1016/j.ejpain.2010.03.007

[32] T. R. Cummins, S. D. Dib-Hajj, S. G. Waxman, Electrophysiological properties of mutant Na_V_1.7 sodium channels in a painful inherited neuropathy. J. Neurosci. **24**, 8232–8236 (2004). 15385606 10.1523/JNEUROSCI.2695-04.2004 PMC6729696

[33] M. T. Wu, P. Y. Huang, C. T. Yen, C. C. Chen, M. J. Lee, A novel SCN9A mutation responsible for primary erythromelalgia and is resistant to the treatment of sodium channel blockers. PLoS One **8**, e55212 (2013). 23383113 10.1371/journal.pone.0055212 PMC3561374

[34] C. M. Kerth, P. Hautvast, J. Körner, A. Lampert, J. E. Meents, Phosphorylation of a chronic pain mutation in the voltage-gated sodium channel Na_V_1.7 increases voltage sensitivity. J. Biol. Chem. **296**, 100227 (2021). 33361158 10.1074/jbc.RA120.014288 PMC7948457

[35] A. Lampert, S. D. Dib-Hajj, L. Tyrrell, S. G. Waxman, Size matters: Erythromelalgia mutation S241T in Na_V_1.7 alters channel gating. J. Biol. Chem. **281**, 36029–36035 (2006). 17008310 10.1074/jbc.M607637200

[36] J. F. Fohlmeister, Voltage gating by molecular subunits of Na^+^ and K^+^ ion channels: Higher-dimensional cubic kinetics, rate constants, and temperature. J. Neurophysiol. **113**, 3759–3777 (2015). 25867741 10.1152/jn.00551.2014 PMC4468971

[37] S. Cestèle , Voltage sensor-trapping: Enhanced activation of sodium channels by beta-scorpion toxin bound to the S3–S4 loop in domain II. Neuron **21**, 919–931 (1998). 9808476 10.1016/s0896-6273(00)80606-6

[38] D. Jiang , Structural basis for voltage-sensor trapping of the cardiac sodium channel by a deathstalker scorpion toxin. Nat. Commun. **12**, 128 (2021). 33397917 10.1038/s41467-020-20078-3 PMC7782738

[39] Y. Y. Vilin, N. Makita, A. L. George, P. C. Ruben, Structural determinants of slow inactivation in human cardiac and skeletal muscle sodium channels. Biophys. J. **77**, 1384–1393 (1999). 10465750 10.1016/S0006-3495(99)76987-0 PMC1300427

[40] J. R. Balser , External pore residue mediates slow inactivation in μ1 rat skeletal muscle sodium channels. J. Physiol. **494**, 431–442 (1996). 8842002 10.1113/jphysiol.1996.sp021503 PMC1160645

[41] B. H. Ong, G. F. Tomaselli, J. R. Balser, A structural rearrangement in the sodium channel pore linked to slow inactivation and use dependence. J. Gen. Physiol. **116**, 653–662 (2000). 11055994 10.1085/jgp.116.5.653 PMC2229478

[42] E. Pavlov , The pore, not cytoplasmic domains, underlies inactivation in a prokaryotic sodium channel. Biophys. J. **89**, 232–242 (2005). 15849254 10.1529/biophysj.104.056994 PMC1366521

[43] Y. Y. Vilin, P. C. Ruben, Slow inactivation in voltage-gated sodium channels: Molecular substrates and contributions to channelopathies. Cell Biochem. Biophys. **35**, 171–190 (2001). 11892790 10.1385/CBB:35:2:171

[44] J. Payandeh, T. M. Gamal El-Din, T. Scheuer, N. Zheng, W. A. Catterall, Crystal structure of a voltage-gated sodium channel in two potentially inactivated states. Nature **486**, 135–139 (2012). 22678296 10.1038/nature11077 PMC3552482

[45] J. Li, J. Ostmeyer, L. G. Cuello, E. Perozo, B. Roux, Rapid constriction of the selectivity filter underlies C-type inactivation in the KcsA potassium channel. J. Gen. Physiol. **150**, 1408–1420 (2018). 30072373 10.1085/jgp.201812082 PMC6168234

[46] A. Lampert, M. Eberhardt, S. G. Waxman, Altered sodium channel gating as molecular basis for pain: Contribution of activation, inactivation, and resurgent currents. Handb. Exp. Pharmacol. **221**, 91–110 (2014). 24737233 10.1007/978-3-642-41588-3_5

[47] Z. Otwinowski, W. Minor, Processing of X-ray diffraction data collected in oscillation mode. Methods Enzymol. **276**, 307–326 (1997). 27754618 10.1016/S0076-6879(97)76066-X

[48] A. J. McCoy , Phaser crystallographic software. J. Appl. Crystallogr. **40**, 658–674 (2007). 19461840 10.1107/S0021889807021206 PMC2483472

[49] G. N. Murshudov , REFMAC5 for the refinement of macromolecular crystal structures. Acta Crystallogr. D Biol. Crystallogr. **67**, 355–367 (2011). 21460454 10.1107/S0907444911001314 PMC3069751

[50] M. D. Winn , Overview of the CCP4 suite and current developments. Acta Crystallogr. D Biol. Crystallogr. **67**, 235–242 (2011). 21460441 10.1107/S0907444910045749 PMC3069738

[51] P. Emsley, B. Lohkamp, W. G. Scott, K. Cowtan, Features and development of Coot. Acta Crystallogr. D Biol. Crystallogr. **66**, 486–501 (2010). 20383002 10.1107/S0907444910007493 PMC2852313

[52] D. Liebschner , Macromolecular structure determination using x-rays, neutrons, and electrons: Recent developments in Phenix. Acta Crystallogr. D Struct. Biol. **75**, 861–877 (2019). 31588918 10.1107/S2059798319011471 PMC6778852

[53] N. Eswar , Comparative protein modeling using modeller. Curr. Protoc. Bioinformatics **54**, 5.6.1–5.6.37 (2016). 10.1002/cpbi.3 PMC5031415 27322406

[54] G. Wisedchaisri, T. M. Gamal El-Din, N. Zheng, W. A. Catterall, Crystal structure of NaVAb I119T as a basis for the human NaV1.7 Inherited Erythromelalgia I234T mutation. Protein Data Bank. https://www.rcsb.org/structure/8DIZ. Deposited 29 June 2022.

[55] G. Wisedchaisri, T. M. Gamal El-Din, N. Zheng, W. A. Catterall, Crystal structure of NaVAb L123T as a basis for the human NaV1.7 Inherited Erythromelalgia I848T mutation. Protein Data Bank. https://www.rcsb.org/structure/8DJ0. Deposited 29 June 2022.

[56] G. Wisedchaisri, T. M. Gamal El-Din, N. Zheng, W. A. Catterall, Crystal structure of NaVAb V126T as a basis for the human NaV1.7 Inherited Erythromelalgia S241T mutation. Protein Data Bank. https://www.rcsb.org/structure/8DJ1. Deposited 29 June 2022.

