# Supplementary material for: Structural basis for severe pain caused by mutations in the S4-S5 linkers of voltage-gated sodium channel NaV1.7
Source: Proc Natl Acad Sci U S A. 2023 Mar 30;120(14):e2219624120. doi: 10.1073/pnas.2219624120 (PMC10083536; doi:10.1073/pnas.2219624120)
Supplement: Supplementary file 1 — Appendix 01 (PDF) [file pnas.2219624120.sapp.pdf]

Supporting Information for

Structural Basis for Severe Pain Caused by Mutations in the S4-S5 Linkers of Voltage-Gated Sodium Channel $Na_V1.7$

Goragot Wisedchaisri[1], Tamer M. Gamal El-Din[1], Ning Zheng[1,2,*], and William A. Catterall[1,*]

This PDF file includes:

Figure S1
Tables S1 to S3
Legends for Movies S1 to S3
SI References

Other supporting materials for this manuscript include the following:

Movies S1 to S3

Supporting Figure

```
                          S4            S4-S5 linker            S5
NaVAb           FEILRVLRVLRLFRLVTAVPQMRKIVSALISVIPGMLSVIALMTLFFYIFAIMATQLFG
NaV1.7-D1       VSALRTFRVLRALKTISVIPGLKTIVGALIQSVKKLSDVMILTVFCLSVFALIGLQLFM
NaV1.7-D2       LSVLRSFRLLRVFKLAKSWPTLNMLIKIIGNSVGALGNLTLVLAIIVFIFAVVGMQLFG
NaV1.7-D3       IKSLRTLRALRPLRALSRFEGMRVVVNALIGAIPSIMNVLLVCLIFWLIFSIMGVNLFA
NaV1.7-D4       FRVIRLARIGRILRLVKGAKGIRTLLFALMMSLPALFNIGLLLFLVMFIYAIFGMSNFA
                .  :*  *  * ::  .    :. ::  :   :  : .:  :  :   ::::.. .  *
```

Figure S1. Multiple Amino Acid Sequence Alignments of Na_VAb and human $Na_V1.7$. Amino acid sequences in the region from S4 to S5 segments are shown.

Supporting Tables

Table S1. IEM Mutations

Domain	Location	Mutation (Na_V1.7)	Construct (Na_VAb)	Origin	Phenotype[a]	References
I	S4-S5	I234T	I199T		IEM	Ahn *et al.*, 2010
I	S4-S5	S241T	V126T	Flemish	Familial IEM[b]	Michiels et al., 2005
II	S4-S5	I848T	L123T	Chinese<br>English	Familial IEM[b]	Drenth et al., 2008<br>Wu *et al.*, 2013<br>Yang et al., 2004

Reference List: (1); (2); (3); (4); (5).
[a]Familial IEM indicates at least two affected family members are known.
[b]Interestingly, the same mutation has been found in Na_V1.4 and Na_V1.5 (corresponding to residue I141V). These mutations lead to myotonia and exercise-induced polymorphic ventricular arrhythmia, respectively, caused by a negative shift in $V_{1/2}$ (6-9).

Table S2. Activation properties of Na_VAb and Na_V1.7 with IEM mutations

Mutation	Na_VAb Δ28			Na_V1.7			Reference
	$V_{1/2}$ (mV)	Δ $V_{1/2}$ (mV)	Slope (k) (mV)	$V_{1/2}$ (mV)	Δ $V_{1/2}$ (mV)	Slope (k) (mV)	
WT	-90 ± 1.5	-	9	-	-	-	Gamal El-Din et al., 2019[b]
I119T (I234T)[a]	-127.6 ± 1.5	-37.5	5.6	-43.1 ± 1.0	-17.9	7.5 ± 0.1	Ahn et al., 2010[c]
L123T (I848T)[a]	-105.5 ± 0.5	-15.5	6.6	-38.4 ± 1.0<br>-29.60 ± 0.50<br>-28.06 ± 5.42	-13.8<br>-7.5<br>-9.8	-<br>5.92 ± 0.43<br>8.99 ± 1.48	Cummins et al., 2004[c]<br>Wu et al., 2013[c]<br>Kerth et al., 2021[c]
V126T (S241T)[a]	-102 ± 0.4	-12	5.6	-34.0 ± 1.1	-8.4	6.52 ± 0.26	Lampert et al., 2006[c]

[a]Residue name and number for Na_V1.7
[b]Reference for Na_VAbΔ28
[c]Reference for human Na_V1.7

Table S3. X-ray data collection and refinement statistics of $Na_VAb\Delta28$ structures with $Na_V1.7$ IEM mutations in the S4-S5 linker

**$Na_VAb\Delta28$ with $Na_V1.7$ IEM mutations**	**I119T (I234T)**[a] PDB: 8DIZ	**L123T (I848T)**[a] PDB: 8DJ0	**V126T (S241T)**[a] PDB: 8DJ1
Space group	I422	I422	I422
Cell dimensions (Å)	a = b = 124.8	a = b = 125.0	a = b = 123.4
	c = 192.4	c = 190.8	c = 191.1
	α =β=γ= 90°	α=β=γ= 90°	α=β=γ= 90°
Wavelength (Å)	1.00	1.00	1.00
Resolution (Å)[b]	50 – 2.75	50 – 2.70	50 – 3.10
	(2.80 – 2.75)	(2.75 – 2.70)	(3.15 – 3.10)
Number of reflections	20,054 (929)	20,400 (746)	13,526 (659)
Completeness (%)	99.4 (93.0)	96.5 (71.4)	98.6 (96.3)
Multiplicity	12.7 (4.6)	12.0 (4.1)	6.0 (5.4)
I/σI	16.5 (0.6)	14.0 (0.4)	12.8 (0.9)
CC½	0.997 (0.405)	1.0 (0.690)	0.994 (0.479)
R_{merge}	0.156 (1.318)[c]	0.148 (1.018)[c]	0.133 (1.917)[c]
R_{pim}	0.044 (0.592)	0.042 (0.429)	0.059 (0.891)
**Refinement**			
Resolution (Å)	50 – 2.75	50 – 2.70	50 – 3.10
No. reflections (work/free)	15,701/849	14,422/761	10,898/561
R_{work} / R_{free}	0.225/0.256	0.207/0.248	0.214/0.260
Number of atoms	2,013	2,207	2,158
B-factor	45.0	45.2	59.3
R.m.s. deviations			
Bond length (Å)	0.008	0.007	0.003
Bond angle (°)	1.055	0.925	0.554
MolProbity			
Overall score	2.4	2.3	1.5
Clashscore	7.5	7.7	5.4
Ramachandran favored (%)	92.8	96.6	96.6
Ramachandran outlier (%)	0.0	0.0	0.0
Rotamer outliers	5.5	8.3	0.0

[a]Residue name and number for $Na_V1.7$
[b]Values in parenthesis are for the highest resolution shell.
[c]Due to anisotropic diffraction

Supporting Movies

Movie S1. Transition of Na_VAb/I119T from the resting state to the activated state from the intracellular view. I119T and Val126 are shown as red and orange sticks, respectively. Ser132’ and Asn211’ from a neighboring subunit are shown as yellow and cyan sticks, respectively. S4 is colored in magenta, the S4-S5 linker in orange, and the pore module S5 to S6 in cyan. Dashes indicate distance in Å.

Movie S2. Transition of Na_VAb/L123T from the resting state to the activated state from the intracellular view. L123T, and Asn211’ from a neighboring subunit are shown as red and cyan sticks, respectively. Dashes indicate distance in Å.

Movie S3. Transition of Na_VAb/V126T from the resting state to the activated state from the intracellular view. V126T, Ile216, and Asn211’ from a neighboring subunit are shown as red, teal, and cyan sticks, respectively. Dashes indicate distance in Å.

References for Supporting Information

1. H. S. Ahn *et al.*, A new Nav1.7 sodium channel mutation I234T in a child with severe pain. *Eur J Pain* **14**, 944-950 (2010).
2. J. J. Michiels, R. H. te Morsche, J. B. Jansen, J. P. Drenth, Autosomal dominant erythermalgia associated with a novel mutation in the voltage-gated sodium channel alpha subunit Nav1.7. *Arch Neurol* **62**, 1587-1590 (2005).
3. J. P. Drenth, R. H. Te Morsche, S. Mansour, P. S. Mortimer, Primary erythermalgia as a sodium channelopathy: screening for SCN9A mutations: exclusion of a causal role of SCN10A and SCN11A. *Arch Dermatol* **144**, 320-324 (2008).
4. M. T. Wu, P. Y. Huang, C. T. Yen, C. C. Chen, M. J. Lee, A novel SCN9A mutation responsible for primary erythromelalgia and is resistant to the treatment of sodium channel blockers. *PLoS One* **8**, e55212 (2013).
5. Y. Yang *et al.*, Mutations in SCN9A, encoding a sodium channel alpha subunit, in patients with primary erythermalgia. *J Med Genet* **41**, 171-174 (2004).
6. M. Y. Amarouch, H. Abriel, Cellular hyper-excitability caused by mutations that alter the activation process of voltage-gated sodium channels. *Front Physiol* **6**, 45 (2015).
7. K. J. Paavonen *et al.*, Response of the QT interval to mental and physical stress in types LQT1 and LQT2 of the long QT syndrome. *Heart* **86**, 39-44 (2001).
8. S. Petitprez *et al.*, A novel dominant mutation of the Nav1.4 alpha-subunit domain I leading to sodium channel myotonia. *Neurology* **71**, 1669-1675 (2008).
9. H. Swan *et al.*, Gain-of-function mutation of the SCN5A gene causes exercise-induced polymorphic ventricular arrhythmias. *Circ Cardiovasc Genet* **7**, 771-781 (2014).
